# Decreasing lifetime prevalence of diabetes-related foot ulcers in Norway: repeated cross-sectional population-based surveys from the HUNT study (1995-2019)

**DOI:** 10.3389/fendo.2024.1354385

**Published:** 2024-04-17

**Authors:** Hilde K. R. Riise, Jannicke Igland, Marit Graue, Anne Haugstvedt, Truls Østbye, Eirik Søfteland, Monica Hermann, Sofia Carlsson, Bjørn Olav Åsvold, Marjolein M. Iversen

**Affiliations:** ^1^ Department of Medicine, Haukeland University Hospital, Bergen, Norway; ^2^ Department of Health and Caring Sciences, Western Norway University of Applied Sciences, Bergen, Norway; ^3^ Department of Global Public Health and Primary Care, University of Bergen, Bergen, Norway; ^4^ Department of Family Medicine and Community Health, Duke University School of Medicine, Durham, NC, United States; ^5^ Department of Clinical Medicine, University of Bergen, Bergen, Norway; ^6^ Institute of Environmental Medicine, Karolinska Institutet, Stockholm, Sweden; ^7^ HUNT Center for Molecular and Clinical Epidemiology, Department of Public Health and Nursing, NTNU – Norwegian University of Science and Technology, Trondheim, Norway; ^8^ Department of Endocrinology, St. Olavs Hospital, Trondheim University Hospital, Trondheim, Norway; ^9^ HUNT Research Center, Department of Public Health and Nursing, NTNU – Norwegian University of Science and Technology, Levanger, Norway

**Keywords:** type 2 diabetes, diabetes-related foot ulcer, diabetes complications, lifetime prevalence, prevalence

## Abstract

**Background and aims:**

Diabetes-related foot ulcers (DFU) are a persistent healthcare challenge, impacting both patients and healthcare systems, with adverse effects on quality of life and productivity. Our primary aim was to examine the trends in lifetime prevalence of DFU, as well as other micro- and macrovascular complications in the Trøndelag Health Study (HUNT) in Norway.

**Methods:**

This study consists of individuals ≥20 years with diabetes participating in the population-based cross-sectional HUNT surveys (1995-2019). Prevalence ratios, comparing the lifetime prevalence of DFU and other relevant micro- and macrovascular complications between the HUNT surveys, were calculated using Poisson regression.

**Results:**

The lifetime prevalence (95% confidence interval (CI)) of a DFU requiring three or more weeks to heal was 11.0% (9.5-12.7) in HUNT2, 7.5% (6.3-8.8) in HUNT3 and 5.3% (4.4-6.3) in HUNT4. The decrease in DFU prevalence from 1995 to 2019 was observed in both men and women, for all age groups, and for both type 1 and type 2 diabetes. The highest lifetime prevalence of DFU was found among those with type 1 diabetes. The decrease in HbA1c from HUNT2 to HUNT4 did not differ between those with and without a DFU. The prevalence of chronic kidney disease (eGFR <60 mL/min/1.73 m^2^ (eGFR categories G3-G5)) increased in both individuals with and without a DFU.

**Conclusion:**

Results from the HUNT surveys show a substantial decline in the lifetime prevalence of DFU from 1995 to 2019.

## Introduction

The International Working Group on the Diabetic Foot (IWGDF) Guidelines define a diabetes-related foot ulcer (DFU) as a foot ulcer in a person with current or previously diagnosed diabetes mellitus, and usually accompanied by peripheral neuropathy and/or peripheral artery disease in the lower extremity (1). DFU is associated with reduced quality of life, increased hospitalization and mortality (2–6), and subsequently, costs to the health care service are high. In fact, surveys in Sweden, where health care cost levels and demographics are comparable to Norway, estimated the total cost of one non-amputation DFU to nearly USD 25,000, rising to more than USD 40,000 in the case of amputation (7). In line with these results a more recent systematic review covering five other European countries has revealed substantial costs associated with the diabetic foot and its complications (8).

The reported prevalence of DFU among people with diabetes in developed countries has varied widely from 16.6% in Belgium, to 1.5% in Australia (4). Zhang and colleagues examined the global disability burden for diabetes-related lower-extremity complications and estimated that, in 2016, 131 million people had diabetes-related lower-extremity complications (1.8% of the population) (9). Given the elevated risks of infection, hospitalization, and amputation, the prevention of DFU and recurrent ulcer is one of the most important challenges in the current approach to diabetic foot disease (10), and updated international standards are striving to improve DFU prevention and care. However, there is limited knowledge with high-quality population-based studies on change in the point prevalence, and/or change in lifetime prevalence (defined as “the proportion of people who have had the disease in question at any point in their life (up to the time of assessment)”) of DFU (2). Although relevant micro- and macrovascular complication rates have declined substantially in high-income countries over the past 30 years (11, 12), it is not known whether this is also the case for DFU. Cardiovascular risk reduction is a major part of diabetes care, and it is likely that peripheral arterial disease and microvascular sclerosis associated with DFU reflects established arterial disease elsewhere in the body (13). A possible decrease in CVD risk factors could impact DFU prevalence.

The Trøndelag Health Study (HUNT) (https://www.ntnu.edu/hunt) is an ongoing population-based study in a large region in Norway. Our primary aim was to examine the trend in lifetime DFU prevalence using data from HUNT2 (1995–1997), HUNT3 (2006-2008), and HUNT4 (2017-2019). Secondly, we aimed to examine the trends in lifetime prevalence of other micro- and macrovascular complications among those with and without a DFU.

## Materials and methods

### The Trøndelag health study

HUNT is an ongoing longitudinal study which includes several comprehensive cross-sectional health surveys conducted at 10-year intervals between 1984 and 2019. All inhabitants ≥20 years old in the Trøndelag region were invited to participate (14). All HUNT studies include extensive questionnaire data, clinical measurements, and samples, collected by specially trained health personnel. Non-fasting blood samples were collected and handled according to appropriate standards. More details on data collection may be found in previous publications (14), and all questionnaires may be found at https://www.ntnu.edu/hunt/data/que.

### Study population

In the current study, we used data from HUNT2, HUNT3, and HUNT4, conducted in 1995-1997, 2006-2008, and 2017-2019. A total of 65,228 (69.5% of those invited) participated in HUNT2, 50,800 (54.1% of those invited) participated in HUNT3, and 56,044 (54.0% of those invited) participated in HUNT4. In addition to completing the main questionnaire, those who reported having diabetes (HUNT2 = 2,028, HUNT3 = 2,264, and HUNT4 = 3,334) received an additional diabetes-specific questionnaire. In HUNT2, HUNT3, and HUNT4, 1,630 (80.4%), 1,824 (80.5%), and 2,393 (71.8%) participants completed the diabetes-specific questionnaire, respectively, and were included in the study population in the current analyses. **Supplementary Figure 1** presents the number of individuals who participated in 1) all three health surveys (HUNT2-HUNT4), 2) both HUNT2 and HUNT3, and 3) both HUNT3 and HUNT4.

### Data material

#### Type of diabetes

Type of diabetes (type 1 or type 2 diabetes) was defined using glutamic acid decarboxylase antibodies (GADA) and age at diabetes diagnosis. In HUNT2 and HUNT3, the unit of measure for GADA was antibody index (ai), while HUNT4 used international units per millimeter (IU/mL) (15). GADA was measured in serum samples and analyzed by immunoprecipitation radioligand assay using translation labelled 3H-GAD65 as a labelled reagent. Type 2 diabetes was defined as GADA <5 IU/mL (<0.08 ai) and age at diagnosis ≥30 years. Type 1 diabetes was defined as GADA ≥5 IU/mL (≥0.08 ai) and/or age at diagnosis <30 years. If data on GADA and/or age at diagnosis were missing, we collected this information from a previous HUNT survey, if available. If the participants only had information on either GADA or age at diagnosis, we used the available information to classify them according to type of diabetes. Information was missing for a total of 20 participants on both GADA and age at diagnosis: 13 participants in HUNT2, 2 in HUNT3, and 5 in HUNT4. These participants were excluded from statistical analyses stratified by type of diabetes. An overview of the percentage of missing data may be found in **Supplementary Table 1**.

### Sociodemographic and health-related variables

Self-reported information on sociodemographic and health-related variables was obtained from the main questionnaires, including the following sociodemographic variables: age at participation, education level (highest achieved education (education <10 years, yes/no)), living alone (yes/no), and occupational status (currently employed, yes/no). The highest attained education level from HUNT2 and HUNT4 was used to classify education level in HUNT3 as this information was missing (not included in the questionnaire).

Information on the following health-related variables was also retrieved: body mass index (BMI), current daily smoking (yes/no), blood pressure (mmHg), use of antihypertensive drugs (yes/no), and physical activity (<1 hour a week). Height and weight were measured by trained personnel and BMI (weight in kilograms divided by the squared value of height in meters) was calculated. Self-reported physical activity was assessed by average hours of light or hard physical activity during one week in the last year (HUNT2), and also, how often they exercised (HUNT3 and HUNT4). By using these data, we categorized physical activity as <1 hour a week and ≥1 hour a week. Blood pressure was measured, by trained personnel, in a sitting position after two minutes of rest. Three measurements were taken, one minute apart, of which the mean value of the second and third measurement was used. Lastly, we retrieved information on the use of insulin and other diabetes medications (yes/no), and on duration of diabetes using (years), year of diagnosis and year of participation.

### Primary outcome

We define lifetime prevalence of DFU as the proportion who have had the disease in question at any point in their life up to the point of the assessment (16), and used the following question from the diabetes-specific questionnaire to assess the self-reported lifetime prevalence: “Did you ever have a foot ulcer that did not heal in three weeks?” (HUNT2 and HUNT3), and “Did you ever have a foot ulcer on a toe, foot or ankle that did not heal in three weeks?” (HUNT4) (16). Lifetime prevalence is distinct from what is referred to as lifetime morbid risk, which is the proportion of people who will eventually develop the disease at some time in their entire life span (17).

### Secondary outcomes

Diabetes eye problems were self-reported by asking “Do you have problems with your vision that your doctor has said are related to your diabetes?” (HUNT2-3), and “Has an ophthalmologist found diabetic changes on the retina/fundus” (HUNT4). Chronic kidney disease was defined as estimated glomerular filtration rate (eGFR) <60 mL/min/1.73 m^2^ (eGFR categories G3-G5), while kidney failure was defined as eGFR <30 mL/min/1.73 m^2^ (eGFR categories G4-G5) (18). eGFR was estimated using the Chronic Kidney Disease Epidemiology Consortium (CKD-EPI) formula (19). 

### Statistical analyses

Sociodemographic status and health characteristic of the study population were described using frequencies and percentages for categorical variables and means and standard deviations (SD) for continuous variables.

The lifetime prevalence of DFU and other complications of diabetes were evaluated using cross-sectional data from all three HUNT surveys and calculated as the proportion of the population with diabetes who, at some point in their life (up until the time of assessment), had ever had a foot ulcer that required more than three weeks to heal. Lifetime prevalence in each survey was calculated for the total sample, by age group (20-49, 50-74, and ≥75 years), sex, and diabetes type with 95% confidence intervals. The lifetime prevalence of other micro- and macrovascular complications was defined as the proportion of the diabetes population who, at some point in life, had (or have) any of the following diseases: diabetes eye problems, stroke, MI, and/or angina pectoris. The prevalence of eGFR was assessed as prevalence at time of participation (point prevalence). Trends in the lifetime prevalence of other micro- and macrovascular complications of diabetes are presented with stratification according to diabetes type and presence of DFU.

Prevalence ratios for HUNT3 vs. HUNT2 and HUNT4 vs. HUNT2 were calculated using modified Poisson regression because of convergence problems with log-binomial regression (20). A binary variable for DFU was used as the dependent variable and a three-category variable for HUNT survey was used as independent variable with HUNT2 as the reference category. Prevalence ratios were obtained by exponentiating the regression coefficients. Clustered robust standard error was used to account for repeated participation. All estimates of the prevalence ratios are presented as 1) unadjusted (Model 1), 2) adjusted for age and sex (Model 2), and 3) adjusted for age, sex, education level, living situation, BMI, systolic blood pressure, smoking, use of insulin, eGFR, and HbA1c (Model 3). Variables in model 3 are known risk factors for DFU and other micro- and macrovascular complications and are considered mediators for the association between year of survey and risk of DFU and complications (2). Multiple imputation by chained equations (MICE) was used to impute missing data (21). Less than 5% was missing for the majority of the variables. In HUNT3, all participants with diabetes were invited to a follow-up study, HbA1c was only measured among those with diabetes who also participated in this follow-up after HUNT3, yielding missing HbA1c values for 40.2% of the participants. Persons with missing HbA1c were significantly older than persons with valid HbA1c-measurements, and missing HbA1c was more frequent among women, those living alone and among smokers, indicating some selection in participation. Given that information on age, gender, living situation and smoking was included in imputation models and adjusted for in regression models we consider missing data to be missing at random (MAR) for all variables, including HbA1c. We imputed missing values for the following variables: DFU, education level, living situation, work situation, BMI, systolic blood pressure, total cholesterol, smoking, use of insulin, eGFR, previous MI, previous angina pectoris and HbA1c. Logistic regression was used as the imputation model for binary variables, and linear regression was used for continuous variables. Age and sex were included as auxiliary variables in the imputation equations in addition to the above- mentioned variables. For each missing value, we obtained 50 imputed values. Convergence of the imputation models were evaluated by visual inspection of trace plots of average of imputed values versus number of imputations (**Supplementary Figure 2**). Plausibility of imputed values for HbA1c was evaluated by distribution plots for each imputed dataset (**Supplementary Figure 3**). Imputed HbA1c values had a plausible range with a slightly higher mean compared to observed values, compatible with a MAR-mechanism where persons with missing values get a higher imputed value because of higher age, higher occurrence of living alone and more current smoking. The level of significance was defined as <0.05 in all analyses.

Analyses were performed using Stata software (StataCorp, 2019, Stata Statistical Software: Release 16, College Station, TX: StataCorp LLC).

### Ethics

This study was approved by the Regional Committees for Medical Research and Health Research Ethics in Norway (74975) and the Norwegian Centre for Research Data (150393). All participants gave written informed consent.

### Results

#### Population characteristics

Sociodemographic status and health characteristics of the total diabetes population in HUNT2, HUNT3, and HUNT4 are shown in **Table 1**. Approximately 85% of the study population was classified as having type 2 diabetes. Age at participation was stable across the three health surveys (66.2, 64.7, and 65.6 years, respectively). Comparing the participants in HUNT4 with those in HUNT2 showed an increase in participants with a higher education level and fewer people reported to be currently smoking. There was a notable decrease in people with systolic blood pressure >140 mmHg, and, at the same time, an increase in the use of antihypertensive drugs from 41.8% to 71.0%. HbA1c decreased from HUNT2 to HUNT4, while there was an increase in participants with BMI >30 (**Table 1**).

**Table 1:** Sociodemographic status and health characteristics among participants with diabetes in HUN2-HUNT4, stratified by diabetes foot ulcer (DFU).

	HUNT2 (1995-1997)			HUNT3 (2006-2008)			HUNT4 (2017-2019)		
	Total	No DFU	DFU	Total	No DFU	DFU	Total	No DFU	DFU
Participants, n (%)	1630 (100)	1339 (89·0)	165 (11·0)	1824 (100)	1620 (92·5)	131 (7·5)	2393 (100)	2196 (94·7)	122 (5·3)
Sociodemographic status									
Age at participation, years, mean (SD)	66·2 (13·6)	65·6 (13·7)	66·9 (14·2)	64·7 (12·3)	64·3 (12·1)	66·5 (14·6)	65·6 (12·6)	65·6 (12·5)	64·3 (14·0)
Women, n (%)	830 (50·9)	680 (50·9)*	68 (41·2)*	874 (47·9)	787 (48·6)*	46 (35·1)*	1069 (44·7)	998 (45·5)*	36 (29·5)*
Living alone, n (%)	645 (39·6)	511 (38·2)	75 (45·5)	651 (35·7)	557 (34·4)*	59 (45·4)*	914 (38·2)	834 (38·0)	53 (43·4)
Mandatory education <10 years, n %	864 (60·4)	708 (59·5)	90 (60·8)	595 (36·6)	521 (35·7)	40 (36·7)	420 (17·6)	384 (17·5)	16 (13·2)
Currently employed, n (%)	435 (26·9)	380 (28·5)	39 (24·1)	623 (34·2)	583 (36·0)*	31 (23·7)*	786 (32·9)	731 (33·3)	32 (26·2)
Lifestyle factors									
BMI, mean (SD)	29·0 (4·8)	28·9 (4·8)	29·3 (5·2)	30·0 (5·1)	29·9 (5·1)	30·4 (5·1)	29·7 (5·1)	29·7 (5·0)*	31·0 (5·5)*
BMI >30, n (%)	578 (36·2)	471 (35·8)	62 (38·8)	777 (43·0)	684 (42·5)	59 (48·0)	1021 (43·0)	928 (42·6)*	59 (49·6)*
Current daily smoking, n (%)	262 (16·8)	220 (17·0)	20 (12·7)	244 (13·9)	210 (13·4)	22 (17·3)	199 (8·4)	175 (8·0)	16 (13·1)
Exercise <1 hour a week, n (%)	401 (33·6)	324 (32·7)	51 (39·5)	448 (25·3)	365 (23·2) **	54 (41·5) **	486 (20·7)	447 (20·8)	25 (21·0)
Diabetes-related characteristics									
Type 2 diabetes n (%)	1329 (82·2)	1095 (82·3*)	124 (76·1)*	1620 (88·9)	1435 (88·7)	113 (86·3)	2042 (85·5)	1880 (85·8)	98 (80·3)
Duration of type 1 diabetes, years, mean (SD)	15·0 (13·7)	14·3 (13·4)*	20·2 (14·6)*	20·7 (14·9)	20·0 (14·6) **	25·6 (14·9) **	21·8 (16·5)	21·7 (16·5)*	24·7 (18·0)*
Duration of type 2 diabetes, years, mean (SD)	7·9 (6·7)	7·8 (6·5)*	10·0 (8·3)*	8·6 (7·4)	8·4 (7·2) **	11·1 (8·9) **	12·2 (9·6)	12·1 (9·4)*	14·7 (10·9)*
HbA1c, mmol/mol, mean (SD)	64·9 (19·6)	64·4 (19·0)*	68·5 (21·6)*	55·5 (13·3)	55·4 (13·2)	58·2 (13·9)	51·5 (11·5)	51·4 (11·4)*	54·5 (12·8)*
HbA1c, %, mean (SD)	8·1 (1·8)	8·0 (1·7)*	8·4 (2·0)*	7·2 (1·2)	7·21 (1·2)	7·5 (1·3)	6·9 (1·1)	6·9 (1·0)*	7·1 (1·2)*
Using insulin, n (%)	525 (32·4)	426 (31·9)*	74 (44·9)*	511 (28·9)	440 (28·0) **	57 (44·9) **	659 (28·9)	591 (28·2) **	54 (46·2) **
Using other diabetes medication, n (%)	665 (41·0)	541 (40·5)	64 (38·8)	1166 (65·6)	1038 (65·7)	85 (65·9)	1658 (70·1)	1530 (70·3)	81 (66·9)
Clinical characteristics									
Using antihypertensive drugs, mean (SD)	678 (41·8)	551 (41·3)	71 (43·6)	1145 (62·8)	1008 (62·2)	85 (64·9)	1539 (71·0)	1406 (70·4)	81 (75·0)
Systolic blood pressure, mm Hg, mean (SD)	154·8 (24·1)	154·6 (24·1)	153·9 (24·3)	138·6 (19·0)	138·5 (18·9)	138·3 (20·8)	136·0 (18·5)	136·2 (18·3)	133·7 (18·6)
Systolic blood pressure >140 mm Hg, n (%)	1174 (72·3)	960 (71·9)	118 (72·0)	814 (44·9)	719 (44·6)	57 (43·9)	924 (38·6)	854 (38·9)	37 (30·3)
Serum cholesterol, mmol/l, mean (SD)	6·2 (1·3)	6·2 (1·3)	6·0 (1·3)	5·0 (1·1)	5·0 (1·1)	4·9 (1·1)	4·6 (1·1)	4·6 (1·1)	4·5 (1·3)
Serum HDL cholesterol, mmol/l, mean (SD)	1·3 (0·4)	1·3 (0·4)*	1·2 (0·4)*	1·2 (0·3)	1·2 (0·3)*	1·2 (0·4)*	1·2 (0·3)	1·2 (0·3) **	1·1 (0·3) **
Serum triglycerides, mmol/l, mean (SD)	2·4 (1·6)	2·4 (1·6)	2·4 (1·3)	2·1 (1·3)	2·1 (1·3)	2·3 (1·2)	2·1 (1·3)	2·1 (1·3)*	2·4 (1·4)*

HbA1c indicates hemoglobin A1c; HDL, high-density lipoprotein; SD, standard deviation; DFU, diabetes-related foot ulcer. A total of 274 participants (4·7%) had missing values on diabetes foot-ulcer. Numbers marked by * indicate statistically significantly associations between those with and without a DFU with p-value < 0·05, numbers marked by ** with p-value < 0·001.

We found that the characteristics of the foot ulcer population in HUNT changed during the study period (**Table 1**). Compared to those without a DFU, the proportion of people with BMI >30 increased more among those with a DFU. Furthermore, in those with a DFU, the percentage of people currently smoking remained stable during the study period. In contrast, the percentage of current smokers was more than halved in those without a DFU. The decrease in HbA1c from HUNT2 to HUNT4 did not differ in those with and without a DFU.

### Trends in the lifetime prevalence of diabetes-related foot ulcers

**Figure 1:**
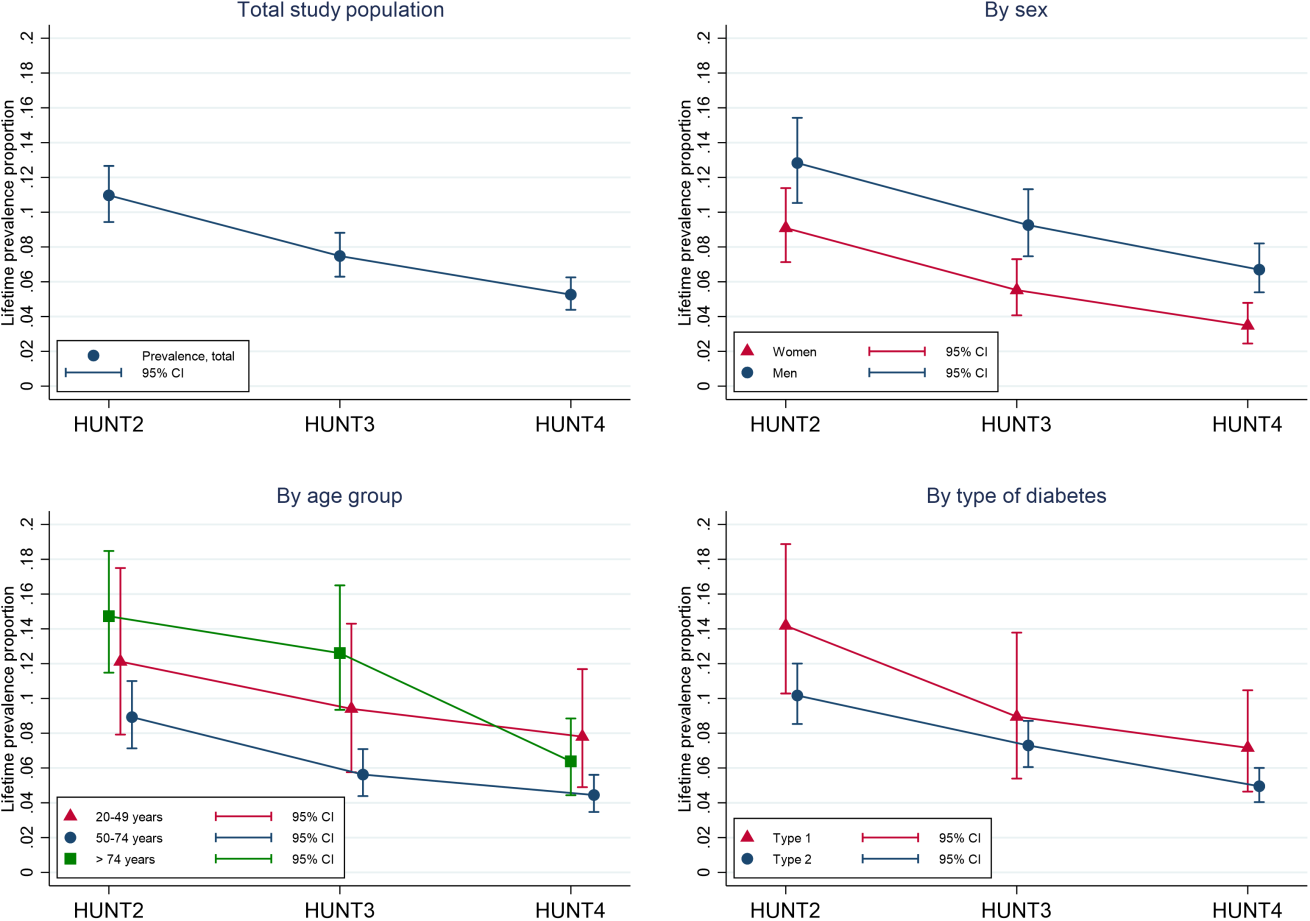
Trends in lifetime prevalence of diabetes-related foot ulcers among participants in HUNT2 (1995-1997), HUNT3 (2006-2008) and HUNT4(2017-2019), in total, and stratified by age, sex and type of diabetes. CI indicates confidence interval.

In the total diabetes population, the lifetime prevalence of a DFU requiring three or more weeks to heal was respectively 11.0% (95% CI: 9.-12.) in HUNT2, 7.5% (95% CI: 6.3-8.8) in HUNT3, and 5.26% (95% CI: 4.4-6.3) in HUNT4 (**Figure 1**, **Table 2**). A total of 165, 131, and 122 participants reported to ever have had a DFU, respectively. The decreasing lifetime prevalence of a DFU was statistically significant at all three measurement points (HUNT2 vs HUNT3, HUNT2 vs HUNT4, and HUNT3 vs. HUNT4). The clear trend of a decreasing lifetime prevalence of DFU from HUNT2 to HUNT4 was observed in both men and women, although men (12.8%, 9.3%, and 6.7%) in general had a higher lifetime prevalence of DFU than women (9.1%, 5.5%, and 3.5%) (p < 0.001). The lifetime prevalence of a DFU decreased in all age groups, with the largest decrease among the oldest (≥75 years). Overall, the lifetime prevalence of a DFU was 30% lower in HUNT3 compared to HUNT2, and 50% lower in HUNT4 compared to HUNT2 (**Table 2**). After adjusting for age, sex, education level, living situation, BMI, systolic blood pressure, smoking, use of insulin, eGFR, and HbA1c using multiple imputation to account for missing values, the estimates did not differ markedly. Results were also similar in complete case analyses (**Supplementary Table 2**).

**Table 2:** Lifetime prevalence (%) and prevalence ratio (PR) of a diabetes-related foot ulcer according to diabetes type in HUNT2-HUNT4, stratified by sex and age.

All participants with diabetes	Lifetime prevalence (95% CI)			Model 1, PR (95% CI)*		Model 2, PR (95% CI) ^†^		Model 3, PR (95% CI) ^‡^	
	HUNT2 (n=1630)	HUNT3 (n=1824)	HUNT4 (n=2393)	HUNT3 vs HUNT2	HUNT4 vs HUNT2	HUNT3 vs HUNT2	HUNT4 vs HUNT2	HUNT3 vs HUNT2	HUNT4 vs HUNT2
*Overall*	11.0 (9·5–12·7)	7·5 (6·3–8·8)	5·3 (4·4–6·23	0·7 (0·6–0·8)	0·5 (0·4–0·6)	0·7 (0·6–0·8)	0·5 (0·4–0·6)	0·7 (0·5–0·8)	0·5 (0·3–0·6)
Sex									
Women	9·1 (7·2–11·4)	5·5 (4·2–7·3)	3·5 (2·5–4·8)	0·6 (0·4–0·9)	0·4 (0·3–0·6)	0·6 (0·4–0·9)	0·4 (0·3–0·6)	0·6 (0·4–0·9)	0·4 (0·2–0·6)
Men	12·8 (10·6–15·4)	9·3 (7·5–11·3)	6·7 (5·5–8·2)	0·7 (0·6–0·9)	0·5 (0·4–0·7)	0·7 (0·6–0·9)	0·5 (0·4–0·7)	0·7 (0·5–0·9)	0·5 (0·4–0·7)
Age groups									
20-49	12·1 (8·3–17·5)	9·4 (6·0–14·2)	7·7 (5·1–11·6)	0·8 (0·4–1·4)	0·6 (0·4–1·1)	0·8 (0·5–1·4)	0·7 (0·4–1·2)	0·9 (0·5–1·6)	0·7 (0·4–1·3)
50-74	8·9 (7·2–11·0)	5·6 (4·5–7·1)	4·5 (3·5–5·6)	0·6 (0·5–0·8)	0·5 (0·4–0·7)	0·6 (0·4–0·8)	0·5 (0·3–0·6)	0·6 (0·4–0·8)	0·5 (0·3–0·6)
≥75	14·7 (11·7–18·4)	12·6 (9·5–16·5)	6·4 (4·6–8·8)	0·9 (0·6–1·2)	0·4 (0·3–0·7)	0·8 (0·6–1·2)	0·4 (0·3–0·6)	0·8 (0·5–1·2)	0·4 (0·2–0·6)
Participants with type 2 diabetes									
	HUNT2 (n=1342)	HUNT3 (n=1622)	HUNT4 (n=2042)	HUNT3 vs HUNT2	HUNT4 vs HUNT2	HUNT3 vs HUNT2	HUNT4 vs HUNT2	HUNT3 vs HUNT2	HUNT4 vs HUNT2
*Overall*	10·2 (8·6–12·0)	7·3 (6·1–8·7)	5·0 (4·1–6·0)	0·7 (0·56–0·91)	0·5 (0·4–0·6)	0·7 (0·6–0·9)	0·5 (0·4–0·6)	0·7 (0·6–0·9)	0·5 (0·3–0·6)
Sex									
Women	8·9 (6·9–11·4)	5·3 (3·9–7·2)	2·9 (2·0–4·3)	0·6 (0·4–0·89)	0·3 (0·2–0·5)	0·6 (0·4–1·0)	0·4 (0·2–0·6)	0·7 (0·4–1·1)	0·4 (0·2–0·7)
Men	11·5 (9·2–14·3)	9·1 (7·3–11·2)	6·5 (5·2–8·1)	0·8 (0·6–1·1)	0·6 (0·4–0·8)	0·8 (0·6–1·1)	0·6 (0·4–0·8)	0·7 (0·5–1·0)	0·5 (0·4–0·8)
Age groups									
20-49	10·2 (5·4–18·5)	7·8 (4·1–14·2)	6·9 (3·7–12·7)	0·8 (0·3–1·8)	0·7 (0·3–1·6)	0·8 (0·3–2·0)	0·8 (0·3–1·8)	1·0 (0·4–2·6)	1·0 (0·4–2·8)
50-74	8·5 (6·7–10·7)	5·6 (4·4–7·1)	4·1 (3·2–5·3)	0·7 (0·5–0·9)	0·5 (0·3–0·7)	0·6 (0·5–0·9)	0·4 (0·3–0·6)	0·6 (0·4–0·9)	0·5 (0·3–0·7)
≥75	13·5 (10·4–17·4)	12·6 (9·5–16·6)	6·7 (4·8–9·3)	0·9 (0·6–1·4)	0·5 (0·3–0·8)	0·9 (0·6–1·3)	0·5 (0·3–0·7)	0·9 (0·6–1·4)	0·4 (0·3–0·8)

PR indicates prevalence ratio; CI, confidence interval.

^*^ Poisson regression without any adjustments (Model 1).

^†^ Poisson regression with adjustment for age and sex (Model 2).

^‡^ Poisson regression with adjustment for age, sex, educational level, living situation, body mass index, systolic blood pressure, smoking, use of insulin, estimated glomerular filtration rate, and HbA1c (Model 3). Missing values in adjustment variables imputed using MICE (Multiple imputation using chained equations).

A total of 274 participants had missing values on diabetes-related foot ulcers (126 in HUNT2, 73 in HUNT3 and 75 in HUNT4).

In individuals with type 2 diabetes, the lifetime prevalence of a DFU was 10.2% (95% CI: 8.7-12.0) in HUNT2, 7.3% (95% CI: 6.2-8.8) in HUNT3, and 5.0.% (95% CI: 4.1-6.0) in HUNT4 (**Table 2**). The trends in DFU lifetime prevalence among men and women and the different age groups were similar to those in the total diabetes population. The prevalence ratio of a DFU was 50% lower in HUNT4 compared to HUNT2: 60% lower in women and 50% lower in men (**Table 2**). Adjustment for known risk factors had minimal impact on the results. The lifetime prevalence of a DFU in those with type 1 diabetes was 14.2% (95% CI: 10.5-18.8), 9.0% (95% CI: 5.6-13.8), and 7.2% (95% CI: 4.8-10.5), respectively (**Figure 1** (numbers not shown in table)).

### Trends in the lifetime prevalence of other micro- and macrovascular diabetes complications

The changes in lifetime prevalence of micro- and macrovascular diabetes complications from HUNT2 to HUNT4 stratified by diabetes-related foot ulcer are reported in **Table 3** and **Supplementary Table 3**. These complications were found to significantly relate to DFU. Those with a DFU reported more diabetes eye problems, kidney disease, stroke, MI, and angina pectoris than those without a DFU. In HUNT4, more than twice as many participants with a DFU reported having diabetes eye problems compared to those without a DFU. The same was seen for stroke. There was no significant trend in changes in micro- or macrovascular diabetes complications among individuals with a DFU from HUNT2 to HUNT4.

**Table 3:** Trends in lifetime prevalence (%) of other micro- and macrovascular diabetes complications in participants in HUNT2-HUNT4, stratified by diabetes-related foot ulcer.

	All participants		
	HUNT2 (n=1630)	HUNT3 (n=1824)	HUNT4 (n=2393)
	Lifetime prevalence (95% CI)	Lifetime prevalence (95% CI)	Lifetime prevalence (95% CI)
Microvascular complications			
Self-reported diabetes eye problems	13·7 (12·0–15·5)	10·5 (9·1–12·0)	10·1 (8·9–11·4)
eGFR <60 mL/min/1.73 m^2^ (eGFR stages G3-G5)	12·5 (10·3–14·2)	11·8 (10·4–13·3)	16·9 (15·4–18·4)
eGFR <30 mL/min/1.73 m^2^ (eGFR stages G4-G5)	1·2 (0·7–1·8)	0·9 (0·5–1·4)	1·3 (0·9–1·8)
Macrovascular complications			
Self-reported stroke	6·2 (5·1–7·5)	7·5 (6·4–8·8)	7·9 (6·9–9·)
Self-reported MI	12·6 (11·1–14·4)	10·8 (9·5–12·3)	12·3 (11·0–13·7)
Self-reported angina pectoris	20·0 (18·1–22·1)	13·0 (11·5–14·6)	9·0 (7·9–10·3)
	No diabetes-related foot ulcer		
	HUNT2 (n=1339)	HUNT3 (n=1620)	HUNT4 (n=2196)
	Lifetime prevalence (95% CI)	Lifetime prevalence (95% CI)	Lifetime prevalence (95% CI)
Microvascular complications			
Self-reported diabetes eye problems	11·8 (10·1–13·7)	9·7 (8·4–11·3)	9·5 (8·3–10·8)
eGFR <60 mL/min/1.73 m^2^ (eGFR stages G3-G5)	11·7 (10·0–13·5)	11·1 (9·7–12·8)	16·6 (15·1–18·2)
eGFR <30 mL/min/1.73 m^2^ (eGFR stages G4-G5)	0·8 (0·4–1·4)	0·7 (0·4–1·2)	1·2 (0·8–1·8)
Macrovascular complications			
Self-reported stroke	5·1 (4·0–6·4)	6·7 (5·6–8·0)	7·4 (6·4–8·7)
Self-reported acute myocardial infarction	12·8 (11·1–14·7)	10·1 (8·8–11·7)	11·9 (10·59–13·41)
Self-reported angina pectoris	19·2 (17·2–21·4)	12·2 (10·7–13·9)	8·9 (7·7–10·2)
	Diabetes-related foot ulcer		
	HUNT2 (n=165)	HUNT3 (n=131)	HUNT4 (n=122)
	Lifetime prevalence (95% CI)	Lifetime prevalence (95% CI)	Lifetime prevalence (95% CI)
Microvascular complications			
Self-reported diabetes eye problems	23·9 (17·8–31·3)	18·9 (13·0–26·7)	20·7 (14·3–28·9)
eGFR <60 mL/min/1.73m2 (eGFR stages G3-G5)	16·4 (11·4–22·9)	16·3 (10·8–23·8)	22·7 (16·0–31·2)
eGFR <30 mL/min/1.73m2 (eGFR stages G4-G5)	3·6 (1·6–7·9)	3·1 (1·2–8·0)	2·5 (0·8–7·6)
Macrovascular complications			
Self-reported stroke	13·1 (8·7–19·3)	17·6 (11·9–25·1)	16·5 (10·6–24·8)
Self-reported acute myocardial infarction	14·8 (10·1–21·2)	15·3 (10·0–22·6)	14·9 (9·4–22·8)
Self-reported angina pectoris	23·5 (17·5–30·7)	19·1 (13·2–26·8)	10·0 (5·6–17·3)

eGFR indicates estimated glomerular filtration rate; CI, confidence interval.

Prevalence of eGFR indicates prevalence at time of participation (point prevalence). eGFR stages according to the NKF-KDOQI guideline for evaluation, classification, and stratification of chronic kidney disease from 2002.

In the total diabetes population, the lifetime prevalence of self-reported eye problems was numerically lower in HUNT2 compared to HUNT4 (13.7% to 10.1%). The prevalence of eGFR <60 mL/min/1.73 m^2^, indicating chronic kidney disease (eGFR stages G3-G5), increased during the same period ((from 12.5% to 16.9%) **Table 3**). Participants in HUNT4 had an almost 40% increased prevalence (PR 1.4 (95% CI 1.1-1.7)) of chronic kidney disease compared to participants in HUNT2 (**Supplementary Table 4**).

Small changes were observed in the lifetime prevalence of MI and stroke from HUNT2 to HUNT4. The lifetime prevalence of angina pectoris changed substantially (from 20.0% to 9.0%), corresponding to a risk reduction of 30% (PR 0.7; 95% CI 0.6-0.8) and 50% (PR 0.5; 95% CI 0.4-0.6) from HUNT2 to HUNT3, and from HUNT2 to HUNT4, respectively. There was an increased lifetime prevalence of stroke in HUNT4 compared to HUNT2 (PR 1.2; 95% CI 0.9-1.6), while the lifetime prevalence of MI remained unchanged (**Table 3**; **Supplementary Table 4**). The same associations were found among those with type 2 diabetes.

## Discussion

In this population-based study from Norway, there was a more than 50% decrease in lifetime prevalence of DFU from 1995 to 2019. The decrease was observed for both men and women, and for all age groups. Decreasing trends in type 1 and type 2 diabetes were similar, but the highest lifetime prevalence of DFU was found among those with type 1 diabetes.

A review by McDermott and colleagues from 2022 (2) highlights the need for high-quality population-based studies of DFU incidence and prevalence. They also state that despite fluctuations in DFU incidence, the patterns of DFU prevalence have remained stable. To our knowledge, this is the first large population-based study with more than two points of measurement documenting a substantial decrease in the lifetime prevalence of DFU over 25 years. Our results align well with a Danish study assessing time trends in DFU incidence (22), which found that the DFU incidence in type 2 diabetes dropped from 8.1% in 2002 to 2.6% in 2014. The corresponding figures for those with type 2 diabetes were 17.0% and 8.7%, respectively. Another Danish study found decreasing incidence trends of distal symmetric polyneuropathy from 1996 to 2018 in both type 1 and type 2 diabetes (23). The results from these two Danish studies are, however, not directly comparable to ours as we report lifetime prevalence and not incidence.

The systematic review by Zhang et al. found higher DFU prevalence in patients with type 2 diabetes (6.4%) than patients with type 1 diabetes (5.5%) (4). This contrasts with the current study where the lifetime prevalence of DFU tended to be higher in type 1 diabetes. Foot ulceration is a major complication of both type 1 and type 2 diabetes, but the higher lifetime prevalence of DFU in type 1 diabetes observed in the current study may be related to the duration of diabetes, which is closely related to age. As expected, mean diabetes duration was higher among those with type 1 diabetes compared to those with type 2 (HUNT2 15.0 vs 7.9 years, HUNT3 20.7 vs 8.6 years, HUNT4 21.8 vs 12.2 years).

During the study period, we found significant improvements in DFU risk factors in the total diabetes population, such as better glycemic and metabolic control. After adjusting for these risk factors, we still observed small changes in DFU lifetime prevalence, indicating that the decrease in DFU prevalence in Norway may not only be explained by enhanced management of DFU risk factors. In line with our results, several recent studies have showed decreasing risk of diabetes risk factors. For instance, a recent publication from Denmark showed a reduction in incidence of neuropathy in both type 1 and type 2 diabetes (23), and a Norwegian study reported moderate improvements in overall control of risk factors such as HbA1c, blood pressure and total cholesterol (24). Nevertheless, advances in clinical and preventive care, as well as improvements in the performance of the health care system may possibly have contributed more to the decrease in DFU prevalence than changes in risk factors. Both national and international guidelines during the last 20 year have been more precise and explicit regarding prevention and treatment of DFU, such as the recommendations for an annual comprehensive foot examinations for all patients with diabetes (2). Furthermore, the increasing prevalence of diabetes (new and existing cases of disease) might also have influenced our results. It is therefore possible that the decrease in the lifetime prevalence of DFU in the current study reflects the larger diabetes population (denominator) with better glycemic and metabolic control.

We found that the prevalence trends of other micro- and macrovascular complications varied during the study period. There was a clear decrease for angina pectoris and an increase of reduced kidney function (eGFR stages G3-G5), which is in line with previous studies (25, 26). The overall mean years lived with diabetes has increased due to a combination of decreasing mortality and increasing diabetes prevalence, which could lead to a range of diabetes co-morbidities, including high rates of renal disease. Overall, those with a DFU in the current study reported a higher prevalence of diabetes eye problems, kidney disease, stroke, MI, and angina pectoris compared to participants without a DFU. This is in line with factors associated with the at-risk foot (27, 28), reflecting the severity of diabetes in the DFU population.

Diabetes-related lower-extremity complication, including DFU, have a significant impact on global disease burden and targeted strategies are needed to help diabetes management, as well a specific DFU strategies such as interdisciplinary foot care services (9). Although a decrease in DFU lifetime prevalence is found in the current study, awareness is needed as the increase in diabetes prevalence and improved survival in people with diabetes can turn the positive trend of decreasing DFU prevalence in Norway. Future research is expected to closely monitor these trends and identify strategies to maintain or further reduce the prevalence of DFUs. Further research may focus on the impact of new treatment approaches, the role of technology in monitoring and early detection of DFUs, and the effectiveness of initiatives intended to raise awareness and improve foot care among individuals with diabetes.

The present study has several strengths. First, these health surveys were large population-based studies. Secondly, we had access to a range of variables on somatic illness, medications, lifestyle, and health-related characteristics measured at three different time points over a period of 25 years. Thirdly, type 1 and type 2 diabetes were verified by GADA measurements (15), and we were consequently able to assess differences in DFU lifetime prevalence between these two types of patients.

However, as with all large-scale-epidemiological studies, this study also has inherent short comings. First, DFU was self-reported and not clinically verified as it was derived from population-based survey data. As such it was not feasible to clinically validate the diagnosis. Self-reported diabetes has shown high validity in HUNT with a specificity of 99.5% and a sensitivity of 81% (14). The specificity of all data related to self-reported cardiovascular disease used in the current study was found to be high (>99%). The sensitivity was lower; 34% for angina pectoris, 76% for acute myocardial infarction, and 59% for heart failure (14). Secondly, the participants in HUNT are recruited from Trøndelag county, a specific geographical area in Norway with few large cities. The population is relatively homogeneous with less than 3% non-Caucasians. Selection bias is a threat for voluntary health surveys in general. Studies examining the non-participants in HUNT [e.g., 15] have shown that they have lower socioeconomic status and have poorer health than the participants, which is in line with other epidemiological studies (29).

## Conclusion

DFU still constitute a substantial burden for people with diabetes. Data from the HUNT Study (1995-2019) allowed changes to be identified in the lifetime prevalence of DFU in people with type 1 and type 2 diabetes. We observed a promising decrease in the lifetime prevalence of DFU over a 25-year period. More studies are needed to investigate this trend in other populations, as well as to elicit the underlying reasons for the decline, with the aim of individualized care further improving outcomes for people with diabetes.

## Data availability statement

The datasets presented in this article are not readily available because the data that support the findings of this study are available from HUNT, but restrictions apply to the availability of these data, which were used under license for the current study and therefore are not publicly available. Anonymous data are however available from the authors upon reasonable request and with permission from HUNT and the Regional Committees for Medical and Health Research Ethics in Norway. Requests to access the datasets should be directed to HR, hkrr@hvl.no.

## Ethics statement

The studies involving humans were approved by Regional Committees for Medical Research and Health Research Ethics in Norway (74975) and the Norwegian Centre for Research Data (150393). The studies were conducted in accordance with the local legislation and institutional requirements. The participants provided their written informed consent to participate in this study.

## Author contributions

HR: Conceptualization, Data curation, Formal analysis, Funding acquisition, Investigation, Methodology, Resources, Software, Supervision, Visualization, Writing – original draft, Writing – review & editing. JI: Data curation, Formal analysis, Methodology, Software, Writing – review & editing. MG: Conceptualization, Funding acquisition, Writing – review & editing. AH: Conceptualization, Funding acquisition, Writing – review & editing. TØ: Writing – review & editing, Conceptualization, Funding acquisition. ES: Writing – review & editing, Conceptualization, Funding acquisition. MH: Conceptualization, Funding acquisition, Writing – review & editing. SC: Data curation, Writing – review & editing. BÅ: Conceptualization, Data curation, Funding acquisition, Writing – review & editing. MI: Conceptualization, Funding acquisition, Project administration, Writing – review & editing.
