# Supplementary material for: Decreasing lifetime prevalence of diabetes-related foot ulcers in Norway: repeated cross-sectional population-based surveys from the HUNT study (1995-2019)
Source: Front Endocrinol (Lausanne). 2024 Apr 17;15:1354385. doi: 10.3389/fendo.2024.1354385 (PMC11061349; doi:10.3389/fendo.2024.1354385)
Supplement: Supplementary file 1 [file DataSheet_1.pdf]

The Trøndelag Health Study (HUNT)

POPULATION

Attendence

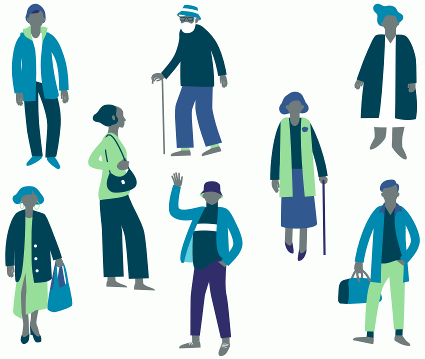

≥ 20 years of age

HUNT2
1995–97

ATTENDED
65,228

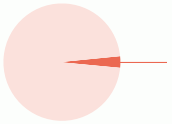

DIABETES
3.1%

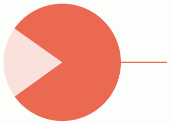

DIABETES-SPESIFIC QUESTIONNAIRE
80.4%
1630 of 2028

HUNT3
2005–07

ATTENDED
50,800

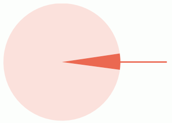

DIABETES
4.5%

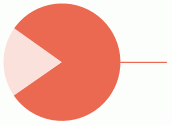

DIABETES-SPESIFIC QUESTIONNAIRE
80.5%
1824 of 2264

HUNT4
2017–19

ATTENDED
56,044

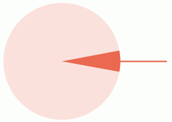

DIABETES
5.9%

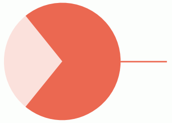

DIABETES-SPESIFIC QUESTIONNAIRE
71.8%
2393 of 3334

METHODS

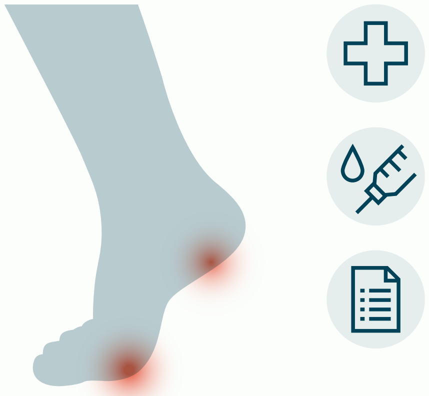

RESULTS

25-year lifetime prevalence of diabetes-related foot ulcers

11.0%
(95% CI: 9.5–12.7)
HUNT2

7.5%
(95% CI: 6.3–8.8)
HUNT3

5.3%
(95% CI 4.4–6.3)
HUNT4

By gender

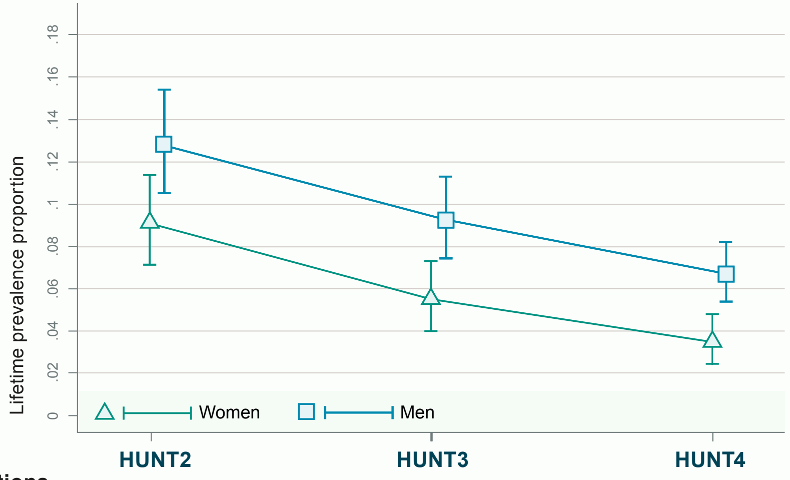

By type of diabetes

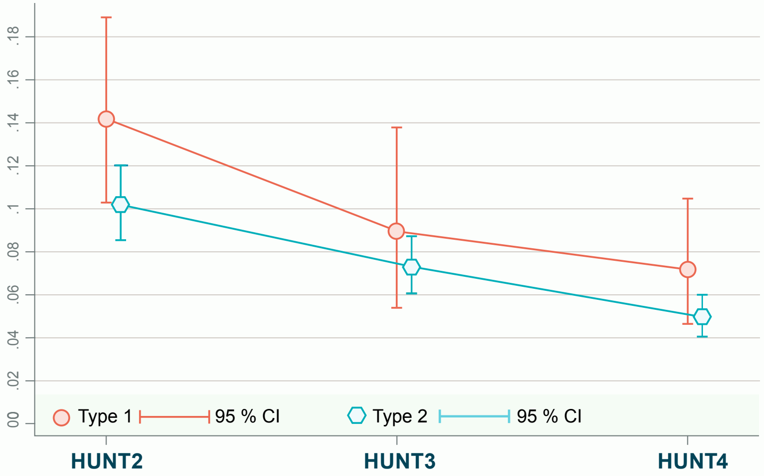

DiaBEST – Best Practice Research in Diabetes and other Chronic Conditions
