# Supplementary material for: Decreasing lifetime prevalence of diabetes-related foot ulcers in Norway: repeated cross-sectional population-based surveys from the HUNT study (1995-2019)
Source: Front Endocrinol (Lausanne). 2024 Apr 17;15:1354385. doi: 10.3389/fendo.2024.1354385 (PMC11061349; doi:10.3389/fendo.2024.1354385)
Supplement: Supplementary file 2 [file DataSheet_2.docx]

**Supplementary Tables and Figures**

**Supplementary Table 1: Overview of missing information on sociodemographic variables and health characteristics in the total diabetes study population.**

|  |  |  |  |
| --- | --- | --- | --- |
|  | **HUNT2 (n=1630)** | **HUNT3 (n=1824)** | **HUNT4 (n=2393)** |
|  |  |  |  |
| **Sociodemographic status** |  |  |  |
| Age at participation, % | 0.0 | 0.0 | 0.0 |
| Women, % | 0.0 | 0.0 | 0.0 |
| Living alone, % | 0.1 | 0.1 | 0.1 |
| Mandatory education, % | 12.3 | 10.9 | 0.2 |
| Currently employed, % | 0.6 | 0.1 | 0.1 |
| **Lifestyle factors** |  |  |  |
| Body mass index, % | 2.1 | 1.0 | 0.9 |
| Current daily smoking, % | 4.2 | 3.7 | 0.8 |
| Exercise <1 hour a week, % | 26.8 | 2.7 | 2.1 |
| **Diabetes-related characteristics** |  |  |  |
| Type of diabetes, % | 0.8 | 0.1 | 0.2 |
| Duration of diabetes, % | 21.0 | 4.2 | 10.1 |
| HbA1c, % | 1.6 | 40.9 | 2.6 |
| Using oral diabetes medication, % | 0.4 | 2.5 | 1.2 |
| Using insulin, % | 0.4 | 3.2 | 4.8 |
| **Clinical characteristics** |  |  |  |
| Using antihypertensive drugs, % | 0.4 | 0.0 | 9.4 |
| Systolic blood pressure, % | 0.3 | 0.5 | 0.0 |
| Serum cholesterol, % | 0.5 | 2.6 | 1.1 |
| Serum HDL cholesterol, % | 1.1 | 2.6 | 0.6 |
| Serum triglycerides, % | 0.5 | 1.3 | 1.1 |
| Estimated glomerular filtration rate | 0.1 | 0.1 | 0.1 |

HbA1c indicates hemoglobin A1c, HDL; high-density lipoprotein.

Missing values in adjustment variables imputed using multiple imputation using chained equations. The following adjustment variables were used: age, sex, educational level, marital status (living alone), body mass index, systolic blood pressure, smoking, use of insulin, estimated glomerular filtration rate, and HbA1c.

.

**Supplementary Table 2: Comparison of prevalence ratios of DFU for complete case analysis versus MICE for the fully adjusted overall log-binomial model in Table 2 (Model 3, upper row).**

|  | **Complete case analysis (n=4270)** | **MICE (n=5847)*** | | | |
| --- | --- | --- | --- | --- | --- |
|  | **PR (95% CI)** | **PR (95% CI)** | **RVI^†^** | **FMI^‡^** | **Relative efficiency^≠^** |
| **Survey** |  |  |  |  |  |
| HUNT2 | 1 (ref.) | 1 (ref.) |  |  |  |
| HUNT3 | 0.68 (0.51-0.92) | 0.67 (0.53-0.85) | 0.09 | 0.08 | 0.998 |
| HUNT4 | 0.45 (0.33-0.61) | 0.45 (0.34-0.59) | 0.09 | 0.08 | 0.998 |
| **Sex** |  |  |  |  |  |
| Women | 1 (ref.) | 1 (ref.) |  |  |  |
| Men | 1.81 (1.42-2.30) | 1.73 (1.41-2.13) | 0.06 | 0.06 | 0.999 |
| **Age** | 1.01 (1.00-1.02) | 1.02 (1.00-1.02) | 0.06 | 0.06 | 0.999 |
| **Low education** |  |  |  |  |  |
| no | 1 (ref.) | 1 (ref.) |  |  |  |
| yes | 0.92 (0.70-1.20) | 0.93 (0.74-1.18) | 0.16 | 0.14 | 0.997 |
| **Marital status** |  |  |  |  |  |
| Unmarried/ divorced/widow | 1 (ref.) | 1 (ref.) |  |  |  |
| Married | 0.73 (0.58-0.92) | 0.70 (0.57-0.84) | 0.08 | 0.08 | 0.998 |
| **BMI** | 1.03 (1.01-1.06) | 1.04 (1.02-1.06) | 0.08 | 0.08 | 0.998 |
| **Systolic blood pressure** | 1.00 (0.99-1.00) | 1.00 (0.99-1.00) | 0.05 | 0.05 | 0.999 |
| **Smoking** |  |  |  |  |  |
| No | 1 (ref.) | 1 (ref.) |  |  |  |
| Yes | 1.16 (0.84-1.60) | 1.15 (0.87-1.52) | 0.11 | 0.10 | 0.998 |
| **HbA1c** | 1.00 (1.00-1.01) | 1.01 (1.00-1.01) | 0.29 | 0.23 | 0.995 |
| **Insulin** |  |  |  |  |  |
| No | 1 (ref.) | 1 (ref.) |  |  |  |
| Yes | 1.66 (1.31-2.08) | 1.71 (1.40-2.10) | 0.10 | 0.09 | 0.998 |
| **eGFR<60 mL/min/1.73m2 (eGFR stages G3-G5)** |  |  |  |  |  |
| No | 1 (ref.) | 1 (ref.) |  |  |  |
| Yes | 1.24 (0.91-1.70) | 1.27 (0.99-1.64) | 0.08 | 0.08 | 0.999 |

^*^MICE: Multiple imputation using chained equations.

^†^RVI: Relative increase in variance. Proportion of increase in variance which is due to missing values.

^‡^FMI: Fraction of Missing Information. Proportion of total variance which is due to missing values.

^≠^Relative efficiency, calculated as 1/(1+FMI/m) where m is the number of imputations.

eGFR stages according to the NKF-KDOQI guideline for evaluation, classification, and stratification of chronic kidney disease from 2002.

**Supplementary Table 3: Trends in lifetime prevalence (%) of other micro- and macrovascular diabetes complications in participants in HUNT2-HUNT4 with type 2 diabetes.**

|  | **Type 2 diabetes** | | |
| --- | --- | --- | --- |
|  | **HUNT2 (n=1329)** | **HUNT3 (n=1620)** | **HUNT4 (n=2042)** |
|  | **Lifetime prevalence 95% CI** | **Lifetime prevalence 95% CI** | **Lifetime prevalence 95% CI** |
| **Microvascular complications** |  |  |  |
| Self-reported diabetes eye problems | 13.3 (11.5-15.3) | 10.3 (8.9-11.9) | 7.9 (6.8-9.2) |
| eGFR <60 mL/min/1.73m2 (eGFR stages G3-G5) | 13.6 (11.9-15.6) | 12.6 (11.1-14.3) | 18.2 (16.6-20.0) |
| eGFR <30 mL/min/1.73m2 | 1.1 (0.7-1.9) | 0.9 (0.5-1.5) | 1.2 (0.9-1.9) |
| **Macrovascular complications** |  |  |  |
| Self-reported stroke | 7.0 (5.7-8.5) | 7.8 (6.6-9.3) | 8.6 (7.4-10.0) |
| Self-reported MI | 13.8 (12.1-15.8) | 11.6 (10.1-13.3) | 13.4 (11.9-15.0) |
| Self-reported angina pectoris | 22.1 (20.0-24.5) | 14.0 (12.4-15.8) | 9.8 (8.5-11.2) |

eGFR indicates, estimated glomerular filtration rate; MI, acute myocardial infarction; CI, confidence interval.

eGFR stages according to the NKF-KDOQI guideline for evaluation, classification, and stratification of chronic kidney disease from 2002.

**Supplementary Table 4: Prevalence ratio (PR) of other micro- and macrovascular diabetes complications in HUNT2-HUNT4, stratified by diabetes type.**

|  | **HUNT3 vs. HUNT2, PR (95% CI)** | | | **HUNT4 vs. HUNT2, PR (95% CI)** | | |
| --- | --- | --- | --- | --- | --- | --- |
|  |  |  |  |  |  |  |
| **All participants with diabetes** |  |  |  |  |  |  |
|  | **Model 1^*^** | **Model 2^†^** | **Model 3^‡^** | **Model 1^*^** | **Model 2^†^** | **Model 3^‡^** |
| **Microvascular complications** |  |  |  |  |  |  |
| Self-reported diabetes eye problems | 0.8 (0.6-0.9) | 0.8 (0.7-0.9) | 0.9 (0.8-1.1) | 0.7 (0.6-0.9) | 0.7 (0.6-0.9) | 0.9 (0.7-1.2) |
| eGFR <60 | 0.9 (0.8-1.1) | 1.1 (1.3-1.7) | 1.0 (0.8-1.2) | 1.4 (1.2-1.6) | 1.5 (1.3-1.7) | 1.4 (1.1-1.7) |
| eGFR <30 | 0.8 (0.4-1.5) | 0.9 (0.4-1.7) | 0.7 (0.4-1.5) | 1.1 (0.6-1.9) | 1.2 (0.6-2.0) | 0.9 (0.4-1.9) |
| **Macrovascular complications** |  |  |  |  |  |  |
| Self-reported stroke | 1.2 (1.0-1.5) | 1.3 (1.0-1.6) | 1.2 (0.9-1.5) | 1.3 (1.0-1.5) | 1.3 (1.0-1.6) | 1.2 (0.9-1.6) |
| Self-reported MI | 0.9 (0.7-1.0) | 0.9 (0.8-1.1) | 0.9 (0.7-1.0) | 1.0 (0.8-1.2) | 0.9 (0.8-1.1) | 01.0 (0.8-1.2) |
| Self-reported angina pectoris | 0.6 (0.6-0.7) | 0.7 (0.6-0.8) | 0.7 (0.6-0.8) | 0.5 (0.4-0.6) | 0.5 (0.4-0.6) | 0.5 (0.4-0.6) |
|  |  |  |  |  |  |  |
| **Participants with type 2 diabetes** |  |  |  |  |  |  |
|  | **Model 1^*^** | **Model 2^†^** | **Model 3^‡^** | **Model 1^*^** | **Model 2^†^** | **Model 3^‡^** |
| **Microvascular complications** |  |  |  |  |  |  |
| Self-reported diabetes eye problems | 0.8 (0.6-0.9) | 0.8 (0.7-1.0) | 0.9 (0.8-1.2) | 0.6 (0.5-0.73) | 0.6 (0.5-0.8) | 0.7 (0.6-1.0) |
| eGFR <60 | 0.9 (0.8-1.1) | 1.1 (0.9-1.3) | 1.0 (0.8-1.2) | 1.3 (1.1-1.58) | 1.5 (1.3-1.7) | 1.4 (1.1-1.7) |
| eGFR <30 | 0.8 (0.4-1.6) | 0.9 (0.4-1.9) | 0.9 (0.4-1.9) | 1.1 (0.6-2.14) | 1.2 (0.7-2.3) | 1.2 (0.5-2.7) |
| **Macrovascular complications** |  |  |  |  |  |  |
| Self-reported stroke | 1.1 (0.9-1.4) | 1.2 (0.9-1.5) | 1.1 (0.8-1.4) | 1.2 (1.0-1.6) | 1.3 (1.0-1.6) | 1.1 (0.8-1.5) |
| Self-reported MI | 0.8 (0.7-1.0) | 0.9 (0.7-1.1) | 0.9 (0.7-1.1) | 1.0 (0.8-1.2) | 0.9 (0.8-1.1) | 1.0 (0.8-1.2) |
| Self-reported angina pectoris | 0.6 (0.5-0.7) | 0.7 (0.6-0.8) | 0.6 (0.6-0.8) | 0.5 (0.4-0.6) | 0.5 (0.4-0.6) | 0.5 (0.4-0.6) |

PR indicates prevalence ratio; eGFR, estimated glomerular filtration rate; MI, acute myocardial infarction; CI, confidence interval.

^*^ Poisson regression without any adjustments (Model 1).

^†^ Poisson regression with adjustment for age and sex (Model 2).

^‡^ Poisson regression with adjustment for age, sex, educational level, marital status, body mass index, systolic blood pressure, smoking, use of insulin, estimated glomerular filtration rate, and HbA1c. Missing values in adjustment variables imputed using MICE (Multiple imputation using chained equations) (Model 3).

**Supplementary Figure 1: Flow chart of the study population in the Trøndelag Health Study (HUNT).**


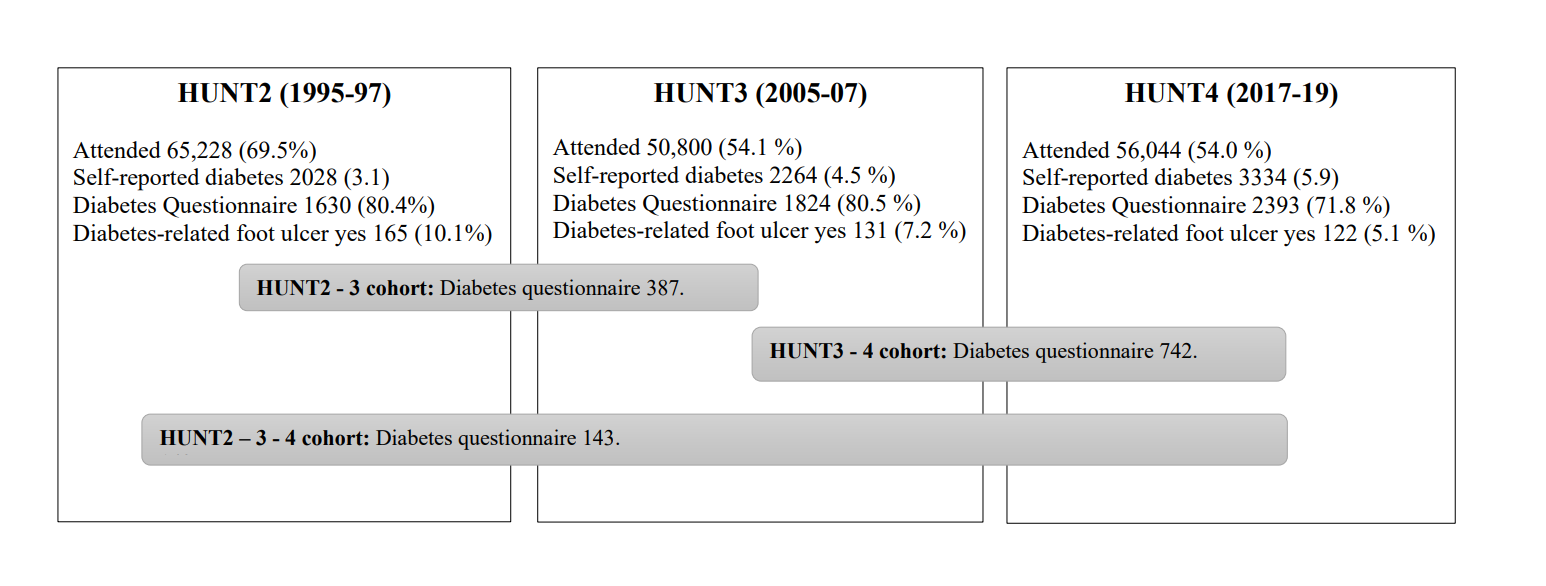


**Supplementary Figure 2: Trace plot of mean and standard deviation for imputed Hba1c-values versus number of iterations for the first three imputed datasets.**

**Supplementary Figure 3: Kernal density plots for the distribution of HbA1c in observed, imputed and completed data for the first 10 imputed datasets.**
